# A Proposed Method for Simultaneous Measurement of Cuticular Transpiration From Different Leaf Surfaces in *Camellia sinensis*

**DOI:** 10.3389/fpls.2020.00420

**Published:** 2020-05-13

**Authors:** Yi Zhang, Xiaobing Chen, Zhenghua Du, Wenjing Zhang, Ananta Raj Devkota, Zijian Chen, Changsong Chen, Weijiang Sun, Mingjie Chen

**Affiliations:** ^1^College of Horticulture and Fujian Provincial Key Laboratory of Haixia Applied Plant Systems Biology, Fujian Agriculture and Forestry University, Fuzhou, China; ^2^Tea Research Institute, Fujian Academy of Agricultural Sciences, Fujian, China; ^3^FAFU-UCR Joint Center for Horticultural Biology and Metabolomics, Haixia Institute of Science and Technology, Fujian Agriculture and Forestry University, Fuzhou, China; ^4^College of Engineering, University of Missouri, Columbia, MO, United States; ^5^Anxi College of Tea Science, Fujian Agriculture and Forestry University, Fuzhou, China; ^6^Henan Key Laboratory of Tea Plant Biology, College of Life Science, Xinyang Normal University, Xinyang, China

**Keywords:** cuticle, cuticular transpiration, epicuticular waxes, intracuticular waxes, adaxial, abaxial, gum arabic, vaseline

## Abstract

The plant cuticle is the major barrier that limits unrestricted water loss and hence plays a critical role in plant drought tolerance. Due to the presence of stomata on the leaf abaxial surface, it is technically challenging to measure abaxial cuticular transpiration. Most of the existing reports were only focused on leaf astomatous adaxial surface, and few data are available regarding abaxial cuticular transpiration. Developing a method that can measure cuticular transpiration from both leaf surfaces simultaneously will improve our understanding about leaf transpiration barrier organization. Here, we developed a new method that enabled the simultaneous measurement of cuticular transpiration rates from the adaxial and abaxial surfaces. The proposed method combined multi-step leaf pretreatments including water equilibration under dark and ABA treatment to close stomata, as well as gum arabic or vaseline application to remove or seal the epicuticular wax layer. Mathematical formulas were established and used to calculate the transpiration rates of individual leaf surfaces from observed experimental data. This method facilitates the simultaneous quantification of cuticular transpiration from adaxial and abaxial leaf surfaces. By applying this method, we demonstrated that the adaxial intracuticular waxes and the abaxial epicuticular waxes constitute the major transpiration barriers in *Camellia sinensis*. Wax analysis indicated that adaxial intracuticular waxes had higher coverage of very long chain fatty acids, 1-alkanol esters, and glycols, which may be attributed to its higher transpiration barrier than that of the abaxial intracuticular waxes.

## Introduction

Global climate change is projected to cause large-scale fluctuations in precipitation patterns, including more frequent incurrences of extreme drought conditions coupled with rising temperature (Scott et al., 2019; https://climate.nasa.gov/effects/). Understanding how plants respond to drought is essential for the development of new germplasms better adapted to the changing environment. Plants have two major pathways for water loss: under normal conditions, stomata transpiration accounts for most of the water loss of plants; under drought conditions, cuticular transpiration becomes the major route for water loss (Holmgren et al., 1965). Thus, cuticular transpiration is tightly associated with plant drought tolerance (Schuster et al., 2017). The plant cuticle covers outer epidermal surface of terrestrial plant species (Ingram and Nawrath, 2017); it minimizes UV irradiance and pollutant entry/retention, defends against pathogens and herbivores, and prevents organ fusion (Jenks et al., 1994; Eigenbrode and Espelie, 1995; Kerstiens, 1996; Chen et al., 2003; Long et al., 2003). However, the principal function of the plant cuticle is to protect leaves, fruits, and other aboveground organs from uncontrolled water loss (Sieber et al., 2000). These diverse functions of the plant cuticle are provided by its complex and heterogeneous chemical and physical nature (Guzmán et al., 2014a). The cutin, cutan, and polysaccharides form polymer matrix as the cuticle backbone (Nawrath, 2006; Domínguez et al., 2011), and cuticular waxes deposit into polymer matrix. Depending on the deposition position, cuticular waxes can be divided into epicuticular waxes (EWs) and intracuticular waxes (IWs). The EWs exist as a thin layer on cuticle’s outer surface; in contrast, the IWs are embedded into cutin/cutan polymer matrix (Jetter et al., 2000). Thus, the EWs can be efficiently stripped off by adhesives such as gum arabic, while IWs are resistant to such treatment (Jetter et al., 2000). Cuticular waxes are complex mixtures of aliphatic (mainly long-chain fatty acids, alcohols, alkanes, aldehydes, esters, and ketones) and aromatic compounds (Jetter et al., 2006). In *Camellia sinensis*, leaf waxes are constituted of 13 chemical classes, including esters, glycols, terpenoids, fatty acids, and their derivatives (Zhu et al., 2018). The cuticle can be divided into three different layers from the external to the internal surface, including the EW layer, the cuticle proper (CP), and the cuticular layer (CL; Jeffree, 2006). It has been traditionally assumed that cutin/cutan and waxes are present in the CP and CL, while polysaccharides are restricted to the CL (Jeffree, 2006). Guzmán et al. (2014a, b, 2016) reported that cellulose and pectins were found on both CP and CL layers. Based on these new findings, Fernández et al. (2016, 2017) proposed that the cuticle may be interpreted as a modified cell wall region which contains additional lipids.

The plant cuticle covers both the adaxial and abaxial leaf surfaces, while stomata are usually present on the abaxial surface. There are lots of interests to investigate cuticular transpiration barrier properties, and several methods have been developed during the past half century. A widely used method is applying microbalance to measure water weight loss from excised leaf. Later, a transpiration chamber measurement method was developed (Becker et al., 1986) and provides more controlled conditions and reproducible results. This method can only be applied with isolated cuticular membranes (CMs) and thus the results may not reflect the transpiration property *in planta*. Different detection techniques have also been developed, including electrolysis cell (Keidel, 1959), microbalance (Schönherr and Lendzian, 1981), moisture sensor, and ^3^H-labeled water in combination with scintillation counter (Schreiber et al., 2001), and the last method offered high precision and sensitivity. CMs isolation method by enzyme digestion was also developed (Schönherr and Riederer, 1986); CMs were isolated from diverse plant species and their transpiration was measured (Becker et al., 1986).

To investigate the cuticular transpiration barrier organization, EWs were selectively stripped off by adhesives such as nitrocellulose, cellulose acetate, or gum arabic (Haas and Rentschler, 1984; Riederer and Schönherr, 1984; Silcox and Holloway, 1986; Jetter and Schäffer, 2001). These studies demonstrated that each adhesive has its own advantages and concerns in terms of convenience, efficiency, and cross-contamination. The transpiration changes after EW removal indirectly reflect the contribution of EWs to the leaf transpiration barrier and directly reflect the contribution of IWs to the leaf transpiration barrier.

The presence of stomata on the leaf abaxial surface makes it technically challenging to measure abaxial cuticular transpiration. It is not surprising that most reports only used astomatous cuticles (Becker et al., 1986; Vogg et al., 2004) and leaf abaxial cuticles were deliberately neglected. To face this challenge, Šantrůček et al. (2004) developed a method that is based on the manipulation of water vapor diffusivity in different types of gases; this method allowed measuring water permeability through stomatous CMs, but only applicable to isolated CMs. Here, we developed a new method to measure cuticular transpiration from the intact leaf. This method can differentiate adaxial transpiration from abaxial transpiration. By applying this method, we demonstrated that adaxial IWs and abaxial EWs constitute the major transpiration barrier in *C. sinensis* cv *Fuyun 6*.

## Materials and Methods

### Reagents

Gum arabic and abscisic acid (ABA) were purchased from Solarbio (Beijing, China), and vaseline was obtained from Borui (Quanzhou, China).

### Plant Material

*Camellia sinensis* cv *Fuyun 6* was grown in a tea garden at Fujian Agriculture and Forestry University (Fuzhou, China; 119.2°E, 26.1°N), and the fourth leaf was used for cuticular transpiration and waxes measurement.

### Leaf EW Removal With Gum Arabic

The fourth leaf from *C. sinensis* cv *Fuyun 6* does not have trichome on either side of the leaf surfaces and was used to localize leaf cuticular transpiration barrier. Gum arabic was selected to remove EWs due to its water solubility, thus avoiding the potential side effects of organic solvents on wax extraction. Before the experiment, gum arabic was extracted seven times with hot chloroform to remove any soluble lipids. A 90% (w/w) aqueous solution of delipidated gum arabic was evenly applied onto leaf surface (∼0.1 ml cm^–2^) with a small soft paintbrush. After 1 h, a dry and stable polymer film was formed, which was peeled off to strip off EWs.

### Leaf Surface Vaseline Sealing

Vaseline was evenly applied onto leaf surface (∼3 mg cm^–2^) with a small soft paintbrush.

### Stomatal Aperture Measurement

Pieces of abaxial epidermal cell layer were peeled off by forceps, mounted onto glass slide with 0.9% NaCl solution, immediately observed under microscope to measure stomatal aperture.

### Leaf Transpiration Measurement

New shoots from clonally propagated *C. sinensis* cv *Fuyun 6* were harvested at the stage of 1 bud with 5–6 leaves at 6:00 pm; the cut ends of the shoots were immersed in fresh tap water and kept in the dark overnight for equilibration. Next day, leaf abaxial surfaces were evenly sprayed with 50 μM ABA, kept in the dark for 1 h, and excess water was gently blotted dry by a soft tissue. Gum arabic or vaseline application was then applied as described above. After completing these pretreatment steps, the fourth leaf was gently removed from each shoot; the excision site on leaf stem was immediately sealed by vaseline. Each leaf was photographed, leaf area (A) was calculated by Image J software, and the initial water saturated fresh weight (W_i_) was recorded; leaves were placed in a controlled dark room (25°C, 45% humidity), and leaf weight was recorded hourly in a balance (JA5003, Liangping, Shanghai, China) for a total 6 h (W_t__1_, _2_,_…__6_). Each treatment group included 4–6 leaves. At the end of experiment, leaves were dried in 80°C for 24 h, and then the dry weight (W_d_) for individual leaf was recorded.

Leaf water loss is given by formula (I):

(I) Leaf water loss = (W_i_ - W_t_)/A
W_i_ and W_t_ represent initial water-saturated leaf weight and leaf weight at t hour post-excision, respectively; A is projected leaf area.

Leaf transpiration rate is given by formula (II):

(II) Leaf transpiration rate = (W_t_ - W_t__+__1_)/A
W_t_, W_t__+__1_ represent leaf weight at t and t + 1 hour post-excision, respectively.

Leaf relative water deficit (RWD) is given by formula (III) (Burghardt (2003):

(III) RWD = [1- (W_t_ - W_d_)/(W_i_ - W_d_)] × 100%

To calculate transpiration rates from the adaxial and abaxial surfaces as well as different cuticle layers, six different treatment groups were prepared simultaneously, including:

(1) Control. The total leaf transpiration rate (T) is given by formula (IV):

(IV) T = T_Ad_ + T_Ab_
T, T_Ad_, and T_Ab_ represent total leaf transpiration rate, adaxial transpiration rate, and abaxial transpiration rate, respectively.

(2) Adaxial surface was sealed with vaseline (Ad/Vas). The observed total transpiration rate (T_Ad__/__Vas_) is given by the formula (V):

(V) T_Ad__/__Vas_ = k x T_Ad_ + T_Ab_
k is vaseline diffusion coefficient factor for water vapor.

(3) Abaxial surface was sealed with vaseline (Ab/Vas). The observed total transpiration rate (T_Ab__/__Vas_) is given by formula (VI):

(VI) T_Ab__/__Vas_ = T_Ad_ + k x T_Ab_

(4) Both leaf surfaces were sealed with vaseline (Ad/Vas:Ab/Vas), the observed total transpiration rate (T_Ad__/__Vas__:__Ab__/__Vas_) is given by formula (VII):

(VII) T_Ad__/__Vas__::__Ab__/__Vas_ = k x (T_Ad_ + T_Ab_)
k is parameter reflecting the physio-chemical nature of vaseline and affected by vaseline film thickness and uniformity. During our experiments, care was taken to ensure that vaseline was evenly applied on both leaf surfaces, and we assume that the vaseline diffusion coefficient factor from both leaf surfaces is identical.

(5) Adaxial EW layer was removed by gum arabic while abaxial surface was sealed with vaseline (-EW_Ad_:Ab/Vas). The observed total transpiration rate (T_–EWAd__:__Ab__/__Vas_) is given by formula (VIII):

(VIII) T_–EWAd__:__Ab__/__Vas_ = T_Ad/intra_ + k x T_Ab_.
T_Ad/intra_ represents the transpiration rate from the adaxial IWs.

(6) Abaxial EW layer was removed by gum arabic while adaxial surface was sealed with vaseline (-EW_Ab_:Ad/Vas). The observed total transpiration rate (T_–EWAb__:__Ad__/__Vas_) is given by formula (IX):

(IX) T_–EWAb__:__Ad__/__Vas_ = T_Ab/intra_ + k x T_ad_.
T_Ab/intra_ represents the transpiration rate from the abaxial IWs.

Adaxial cuticular transpiration can be regarded as the same as T_Ad_. However, due to the possible existence of residual stomatal transpiration from the abaxial leaf surface (T_Ab_s_), abaxial cuticular transpiration (T_Ab_c_) should be lower than T_Ab_. Their relation is given by formula (X):

(X) T_Ab_ = T_Ab_c_ + T_Ab_s_

### Wax Sampling

The shoots with concurrent bud break were selected, and the fourth leaf from each shoot was harvested. Twenty leaves were randomly selected and pooled together as one biological replicate; four biological replicates were used. EWs were removed by gum arabic; the film was collected into a glass tube containing 21 ml of chloroform:water (2:1, v/v), and 75 μg of *n*-tetracosane (Sigma-Aldrich, St. Louis, United States) was added as internal standard. After vigorous agitation and phase separation, the organic phase was transferred into a new glass tube. Extraction was repeated with another 4.5 ml of extraction buffer. The organic phases were combined and evaporated under CentriVap Console (Labconco, KS, United States). The adaxial EWs were firstly removed and then abaxial EWs were isolated from the same batch of leaves.

After EW removal from both leaf surfaces by gum arabic, the leaves were still physically intact, and were used to extract IWs. The adaxial IWs were rinsed five times by chloroform; the elution was collected into a glass beaker. Abaxial IWs were isolated in the same manner. The elution was dried down by CentriVap Console to recover IWs.

### Adaxial and Abaxial IW Analysis

Before analysis, hydroxyl groups in wax lipids were converted to trimethylsilyl derivatives by reacting with *N*,*O*-bis(trimethylsilyl)-trifluoroacetamide (GC grade BSTFA, Aldrich) containing 1% trimethylchlorosilane (Aldrich) in pyridine (Aldrich, 99.8%, anhydrous) and the reaction was maintained at 70°C for 1 h. Each sample was divided into two parts and analyzed by GC-MS (GCMS-QP2010 Ultra, Shimadzu, Japan) and GC-FID (GC-2010 plus, Shimadzu, Japan), respectively. GC-MS and GC-FID were equipped with the same type of capillary column (DB-1, 30 m × 0.25 mm × 0.25 μm, Agilent, California, United States). Helium was used as carrier gas at a constant flow of 1.2 ml min^–1^ and 1.7 ml min^–1^ for GC-MS and GC-FID, respectively. The flow rates for hydrogen, nitrogen, and zero air were 40, 30, and 400 ml min^–1^, respectively. Oven temperature was programmed at 70°C, raised by 10°C min^–1^ to 200°C, held for 2 min, raised by 3°C min^–1^ to 320°C, and held for 20 min. The MS detector setting was as follows: EI-70 eV, ionization source temperature 230°C. Individual wax component was identified by comparing its mass spectra with those of authentic standards and literature data. FID data were used to quantify wax amount by normalizing peak areas from individual wax homologs against that of the internal standard.

### Scanning Electron Microscopy (SEM)

EWs from half of the leaf were stripped by gum arabic; the other half was left untreated as control. Samples were air dried at room temperature. Before observation, small pieces of samples were fixed to aluminum sample holders, freeze dried (HCP-2 critical point dryer, Hitachi, Japan), sputtered with a thin layer of gold (IB5 ion coater, Eiko, Japan), and then observed under scanning electron microscope (SEM) (JEM-6380LV, JEOL, Japan).

### Transmission Electron Microscopy (TEM)

Samples were cut into small pieces (2 mm × 4 mm) and fixed in 5% (v/v) glutaraldehyde solution overnight in 4°C. Samples were rinsed three times with 0.1 M PBS buffer (pH 7.2), post fixed with 1% (w/v) osmium tetroxide at 4°C for 2–2.5 h, rinsed three times with PBS buffer, and then dehydrated through 30% (v/v) and 50% (v/v) ethanol for 15 min. Samples were stained with saturated uranyl acetate overnight, and then rinsed with 70% (v/v) ethanol several times to remove unbound uranyl acetate. Samples were dehydrated in 90% (v/v) and 100% ethanol for 17 min, and dehydrated once more in 100% ethanol for 17 min. Samples were treated sequentially with acetone:ethanol (1:3, v/v), acetone:ethanol (1:1, v/v), acetone:ethanol (3:1, v/v), and 100% acetone for 17 min. The dehydration step was repeated in 100% acetone. Samples were infiltrated through a graded acetone/Epon/Spurr’s epoxy resin and polymerized at 70°C for 24 h (Zhu et al., 2018). Samples were sectioned at 70 nm thickness using an ultra 35° diatome diamond knife; the thin sections were collected onto 200-mesh copper thin bar grids and observed under transmission electron microscope (TEM) (HT7700, Hitachi, Japan).

### Statistical Analysis

Mean and standard error were calculated with ANOVA within standard Excel software package. Probabilities for significance were calculated using Student’s *t* test.

## Results

### Gum Arabic Can Selectively Remove EWs

**FIGURE 1:**
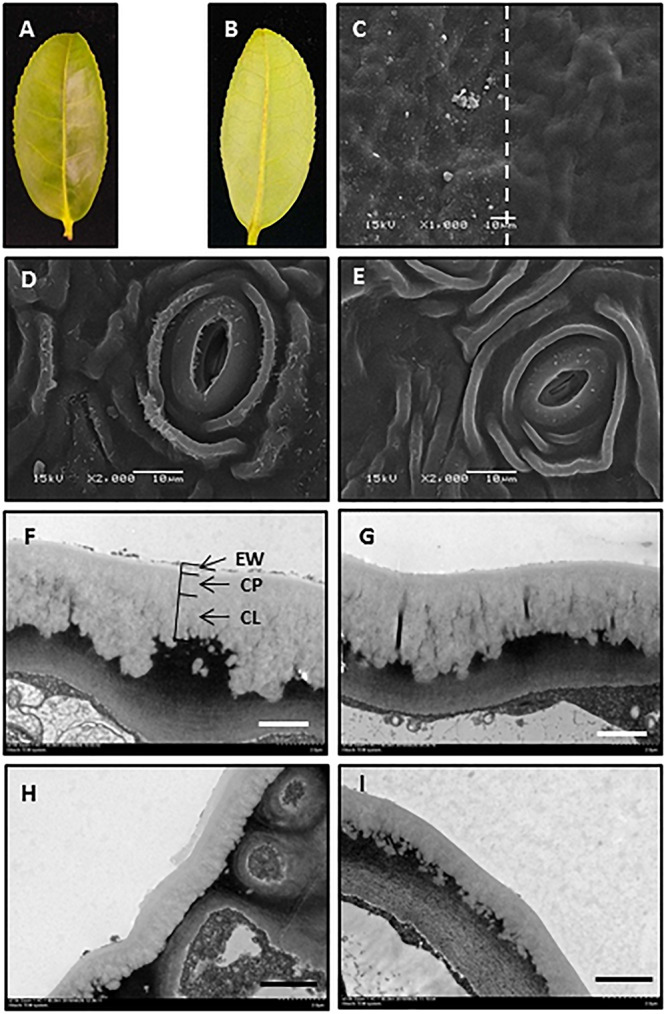
Gum arabic effectively removed epicuticular waxes without affecting intracuticular waxes. **(A,B)** Adaxial and abaxial surfaces of the fourth tea leaf. **(C)** Scanning electron micrograph of adaxial leaf surface before (left) and after (right) gum arabic treatment. **(D,E)** Scanning electron micrograph of abaxial leaf surface before and after gum arabic treatment, respectively. **(F,G)** Transmission electron micrograph of adaxial leaf surface before and after gum arabic treatment, respectively. **(H,I)** Transmission electron micrograph of abaxial leaf surface before and after gum arabic treatment, respectively. Bar = 10.0 μm for (**CߝE**); bar = 2.0 μm for (**FߝI**).

The adaxial and abaxial leaf surfaces looked flat (Figures 1A,B). The mechanical removal of EWs by gum arabic was visualized under SEM and TEM. The native leaf adaxial surface had an irregular coverage with EW crystalloids. When gum arabic was carefully applied onto half of the leaf (Figure 1C, right half), leaving another half untreated (Figure 1C, left half), a clear border between treated and untreated halves of the cuticle was visible (Figure 1C, dashed line). After gum arabic treatment, EW crystals completely disappeared and cuticle exhibited a very smooth appearance (Figure 1C, right half). The native abaxial leaf surface showed ridges and grooves and spread with rod-like crystalloids (Figure 1D). After gum arabic stripping, abaxial EW crystals almost completely disappeared and the cuticle exhibited a smooth appearance (Figure 1E). Stomata kept intact after gum arabic stripping (Figures 1D,E). The cross section of the cuticle before and after gum arabic stripping also was visualized by TEM. Without gum arabic treatment, the outmost cuticle exhibited a rough appearance (Figures 1F–H) and became very smooth after gum arabic treatment (Figures 1G–I). The IW was not affected by gum arabic treatment (Figures 1F–I). By comparing the cuticle thickness difference before and after gum arabic treatment, the thickness of the adaxial and abaxial EWs was estimated to be 0.21 ± 0.03 μm and 0.15 ± 0.02 μm, respectively. Since acetone and ethanol may remove some of the EWs during sample preparation, the actual EW thickness could be larger than the observed value. These observations demonstrated that gum arabic can selectively remove EWs from tea leaves without affecting IWs.

### Gum Arabic Treatment Did Not Disrupt Stomatal Closure

**FIGURE 2:**
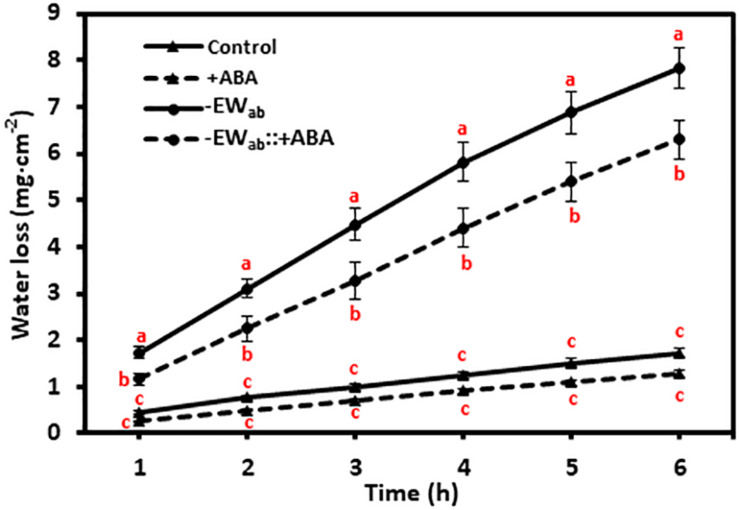
Gum arabic treatment on abaxial surface did not affect stomata closure. Control, leaves not treated with ABA and gum arabic; + ABA, leaves treated with ABA only; -EW_ab_, leaves without ABA pretreatment but treated with gum arabic; -EW_ab_: + ABA, leaves treated with ABA and gum arabic. Data are expressed as means ± standard error (*n* = 5). Statistical analysis was performed among different treatments at same time point, and different letters at the same time point indicate statistically significant (*p* < 0.05).

To test whether gum arabic treatment on leaf abaxial surface affected stomatal closure, leaves were divided into two groups: one group pretreated with ABA (ABA treatment group), and the others left untreated (ABA treatment control group). Then, each group was divided into two subgroups; one subgroup had the leaf abaxial surfaces stripped by gum arabic (gum arabic treatment group) while the second subgroup was left untreated as gum arabic treatment control, and water loss was measured in both cases. If gum arabic treatment disrupted stomata closure, one would expect that after gum arabic treatment, water loss from ABA pretreated leaves would become indistinguishable from the leaves without ABA pretreatment. When leaves were not stripped by gum arabic, water loss from ABA pretreated leaves was lower compared to leaves without ABA treatment, but there was not statistically significant difference at each time point. These observations indicated that ABA application had minor influence on leaf water loss when leaves were fully water equilibrated, and ABA is not sufficient to close stomata under this scenario. After gum arabic stripping, water loss was significantly increased regardless of the pretreatment with ABA or not. However, water loss from ABA-pretreated leaves was significantly lower compared with leaves without ABA treatment (Figure 2); thus, gum arabic stripping did not disrupt stomata closure.

### Vaseline Effectively Reduced Leaf Water Loss

**FIGURE 3:**
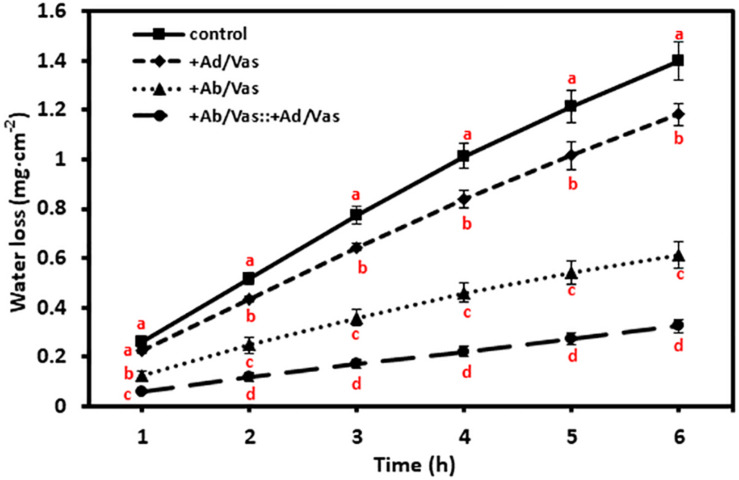
Vaseline application reduced leaf water transpiration. +Ad/Vas, leaf adaxial surface sealed with vaseline; +Ab/Vas, leaf abaxial surface sealed with vaseline; +Ab/Vas: +Ad/Vas, both leaf surfaces sealed with vaseline. Data were expressed as means ± standard error (*n* = 5). Statistical analysis was performed among different treatments at the same time point, and different letters at the same time point indicate statistically significant (*p* < 0.05).

To test the moisture reduction efficiency of vaseline, vaseline was evenly applied to either the adaxial or abaxial surfaces or both surfaces, and leaf water losses were quantified over a 6-h period. We found that vaseline coating on adaxial surface, abaxial surface, and both leaf surfaces significantly reduced water loss by 15%, 56%, and 77%, respectively (Figure 3). These data demonstrated that more water was lost through abaxial surface than that of adaxial surface, and vaseline application can effectively reduce leaf water loss.

### Adaxial IWs and Abaxial EWs Are Respectively the Major Leaf Transpiration Barriers

**FIGURE 4:**
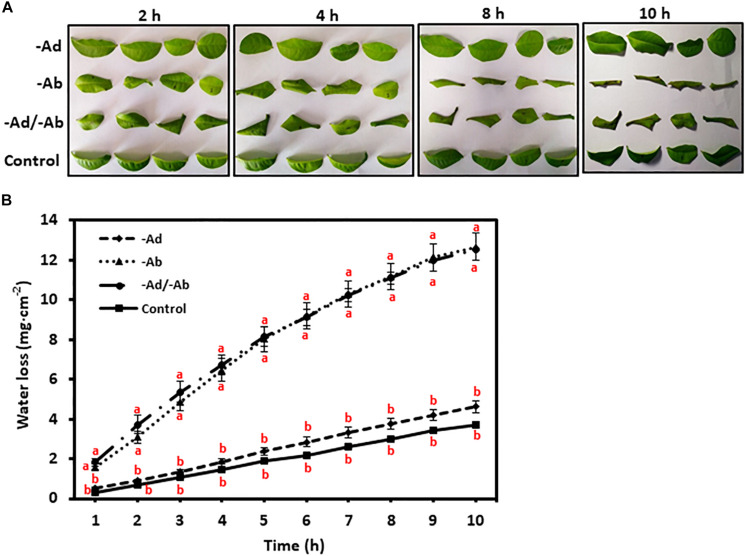
Adaxial and abaxial epicuticular waxes had different contribution to leaf transpiration barrier. -Ad, adaxial epicuticular waxes were removed by gum Arabic; -Ab, abaxial epicuticular waxes were removed by gum Arabic; -Ad/-Ab, epicuticular waxes from both surfaces were removed by gum arabic. Data are expressed as means ± standard error (*n* = 5). Statistical analysis was performed among different treatments at same time point, and different letters at same time point indicate statistically significant (*p* < 0.05). **(A)** Leaf morphological changes after epicuticular wax removal by gum arabic; **(B)** Leaf water loss was affected by the epicuticular wax removal.

To investigate tea leaf transpiration barrier organization, the EWs from the adaxial and abaxial surfaces were removed by gum arabic stripping. When the adaxial EWs were removed, the water loss showed small and statistically insignificant increase compared to untreated control (Figures 4A,B). Thus, our observation in tea leaves was in accordance with previous results from other plant species (Zeisler and Schreiber, 2016). However, when the abaxial EWs were removed, the water loss was significantly increased compared to untreated control (Figures 4A,B). When both adaxial and abaxial EWs were removed simultaneously, the water loss was not significantly different from leaves with abaxial EWs removal only (Figures 4A,B). These observations demonstrated that abaxial EWs were the major transpiration barrier while the adaxial IWs constituted another transpiration barrier.

### Transpiration Rate Measurement of Adaxial and Abaxial Leaf Surfaces

**FIGURE 5:**
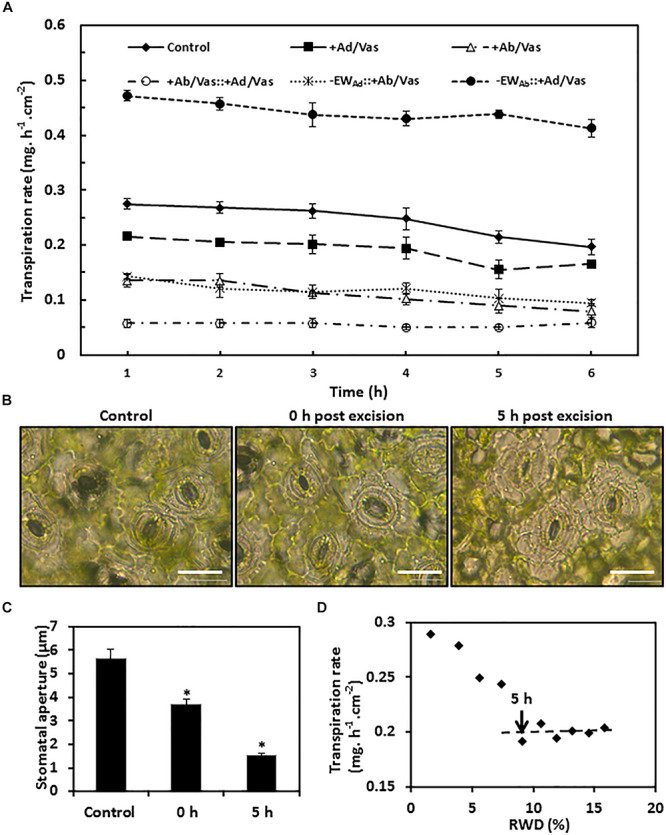
Observed transpiration rate measurement and stomata changes during post-excision. **(A)** Observed transpiration rates during 6 h post-excision. +Ad/Vas, leaf adaxial surface sealed with vaseline; +Ab/Vas, leaf abaxial surface sealed with vaseline; +Ab/Vas: +Ad/Vas, both leaf surfaces sealed with vaseline; -EW_Ad_: +Ab/Vas, adaxial epicuticular waxes removed by gum arabic and abaxial surface sealed with vaseline; -EW_Ab_: +Ad/Vas, abaxial epicuticular waxes removed by gum arabic and adaxial surface sealed with vaseline. Data were expressed as mean ± standard error (*n* = 6). Statistical analysis was performed among different treatments at the same time point, and different letters at the same time point indicate statistically significant (*p* < 0.05). **(B)** Stomata changes before ABA treatment (control), and post-excision (0 h and 5 h). Abaxial epidermal cell layer was removed, mounted onto glass slide, and immersed in 0.9% NaCl solution, then immediately observed under microscope. Bar = 20 μm. **(C)** Stomata aperture before ABA treatment, and post-excision (0 and 5 h). Data were given as mean ± standard error (*n* = 20). Asterisks indicate statistically significant at 0.01 levels. **(D)** The regression curve between leaf transpiration rates and relative water deficit (RWD) during 10 h post-excision.

To measure water transpiration rate from individual leaf surfaces, six different treatments were conducted simultaneously as detailed in the “Materials and Methods” section. In order to calibrate cuticular transpiration, it is essential to estimate residual stomatal transpiration. Two data sets were used for this purpose, including control (leaves without gum arabic and vaseline treatment) and leaves with adaxial surface coated by vaseline. The transpiration rates of the control leaves were kept constant during the first 4 h post-excision, and then showed a small but significant drop at 5 h post-excision (Figure 5A). To identify the source responsible for this transpiration decline, stomata aperture was measured before ABA pretreatment, 0 h, and 5 h post-excision. We found that stomata aperture was reduced 34% by ABA treatment compared with untreated control and was further reduced 59% at 5 h post-excision (Figures 5B,C). After excision, leaf water continuously evaporated, which would reduce the overall leaf water content (Clarke et al., 1991). To investigate how the decline of leaf water content affect stomatal transpiration and cuticular transpiration, leaf transpiration rates and corresponding relative water deficit (RWD) were calculated over 10 h post-excision. We found that for the first 4 h post-excision, the decline in transpiration rates were correlated with RWD increase; starting at 5 h post-excision, the transpiration rate was kept constant regardless of RWD increase (Figure 5D). Burghardt and Riederer (2003) also reported that after complete stomatal closure, a constant minimum transpiration rate was maintained over a wide range of water deficits. These data suggest that stomata closure was responsible for the transpiration drop at 5 h post-excision (Figure 5A), while cuticular transpiration was not affected by the reduction of leaf water content. Thus, the transpiration difference between the 5th h and the 1st h can be used to estimate residual stomatal transpiration (T_Ab_s_); this led to the estimation of residual stomatal transpiration rate as 0.06 ± 0.008 mg h^–1^ cm^–2^, which account for 22% of total leaf transpiration. When the adaxial leaf surface was sealed by vaseline, the residual stomatal transpiration accounted for 28% of the observed total leaf transpiration. When the leaf abaxial surface was sealed by vaseline, the observed transpiration rate was reduced by 0.13–0.15 mg. h^–1^ cm^–2^ compared to that of control leaves. Residual stomatal transpiration may be largely eliminated by vaseline coating on the abaxial surface; abaxial cuticular transpiration was thus reduced in the range of 0.07–0.09 mg h^–1^ cm^–2^ by vaseline coating (Figure 5A).

When the abaxial EWs were removed by gum arabic and the adaxial surface was sealed by vaseline, the observed transpiration rate drop was observed at 4 h post-excision (Figure 5A), and the transpiration difference between the 4th h and the 1st h was 0.042 ± 0.006 mg h^–1^ cm^–2^, which was close to the value calculated from control leaves (0.06 ± 0.008 mg h^–1^ cm^–2^). These data further demonstrated that gum arabic stripping on leaf abaxial surface did not affect stomata closure.

Based on the observed transpiration rates and the estimated residual stomatal transpiration rates, the transpiration rates from adaxial surface, adaxial IWs, abaxial surface, abaxial cuticle, and abaxial IWs were calculated according to their mathematical relationships in formula IV–IX (Table 1). Since leaf water content was continuously decreasing after excision, the transpiration rate at the first hour post-excision would offer a more accurate estimation. For comparison, the hourly transpiration rates during the 6-h post-excision are listed (Table 1). Vaseline diffusion coefficient factor (k), which is expressed as the ratio of transpiration with both leaf surfaces sealed by vaseline to that of control leaves, gradually increased from 0.21 to 0.29 during the 6-h post-excision; this mild increase mainly resulted from the gradual decrease of transpiration rate in the control (Figure 5A). The transpiration rates from either leaf surfaces or IWs showed general downward trends (Table 1); this was attributed to the k increase during post-excision. The transpiration rate from abaxial surface (T_Ab_) was about 1-fold higher than that of adaxial surface (T_Ad_), residual stomatal transpiration (T_Ab_S_) accounted for one third of abaxial transpiration (T_Ab_), and the remaining two thirds should be attributed to abaxial cuticular transpiration (T_Ab_C_). After excluding residual stomatal transpiration, the abaxial cuticular transpiration rate was still about 50% higher than that of adaxial surface. When adaxial EWs were removed by gum arabic, the transpiration rate from adaxial IW (T_Ad/intra_) remained almost unchanged as that of control leaves (T_Ad_), indicating that adaxial EWs did not contribute much to the transpiration barrier. However, when abaxial EWs were removed by gum arabic, the abaxial IW transpiration rate (T_Ab/intra_c_) was more than onefold higher than that of control leaves (T_Ab_C_), and was threefolds higher than that of adaxial IW (Table 1). These data further demonstrated that the adaxial IWs and the abaxial EWs were the major leaf transpiration barriers, while adaxial EWs and abaxial IWs contributed minor to the cuticular transpiration barriers in *C. sinensis*.

**TABLE 1:** Water transpiration rates from different leaf sides and different cuticle layers.

	**K**	**T_Ad_**	**T_Ab_**	**T_Ab–C_**	**T_Ad/intra_**	**T_Ab/intra_**	**T_Ab/intra–C_**
		**(mg h^–1^.cm^–2^)**	**(mg h^–1^.cm^–2^)**	**(mg h^–1^.cm^–2^)**	**(mg h^–1^.cm^–2^)**	**(mg h^–1^.cm^–2^)**	**(mg h^–1^.cm^–2^)**
1 h	0.206 ± 0.019	0.094 ± 0.011^e^	0.199 ± 0.006^c^	0.142 ± 0.002^d^	0.104 ± 0.002^e^	0.452 ± 0.008^a^	0.413 ± 0.004^b^
2 h	0.211 ± 0.022	0.095 ± 0.012^e^	0.190 ± 0.005^c^	0.133 ± 0.003^d^	0.097 ± 0.005^e^	0.437 ± 0.009^a^	0.398 ± 0.003^b^
3 h	0.224 ± 0.035	0.069 ± 0.003^d^	0.198 ± 0.017^b^	0.141 ± 0.013^c^	0.077 ± 0.010^d^	0.422 ± 0.022^a^	0.383 ± 0.017^a^
4 h	0.213 ± 0.036	0.061 ± 0.007^d^	0.189 ± 0.023^b^	0.132 ± 0.019^c^	0.087 ± 0.005^d^	0.417 ± 0.012^a^	0.378 ± 0.006^a^
5 h	0.238 ± 0.031	0.053 ± 0.010^e^	0.154 ± 0.017^c^	0.096 ± 0.013^d^	0.078 ± 0.013^de^	0.426 ± 0.006^a^	0.387 ± 0.004^b^
6 h	0.293 ± 0.034	0.029 ± 0.014^e^	0.172 ± 0.005^c^	0.115 ± 0.008^d^	0.048 ± 0.006^e^	0.405 ± 0.012^a^	0.365 ± 0.006^b^

*K: Vaseline diffusion coefficient; T_*Ad*_: adaxial cuticular transpiration rate; T_*Ab*_: abaxial transpiration rate; T_*Ab–C*_: abaxial cuticular transpiration rate; T_*Ad/intra*_: adaxial intracuticular transpiration rate; T_*Ab/intra*_: abaxial intra transpiration rate; T_*Ab/intra–C*_: abaxial intracuticular transpiration rate. Data are given as means ± standard error (*n* = 6). The different letters in the same row indicate significant difference among treatments at the 0.05 level.*

### Adaxial and Abaxial IW Compositional Analysis

**FIGURE 6:**
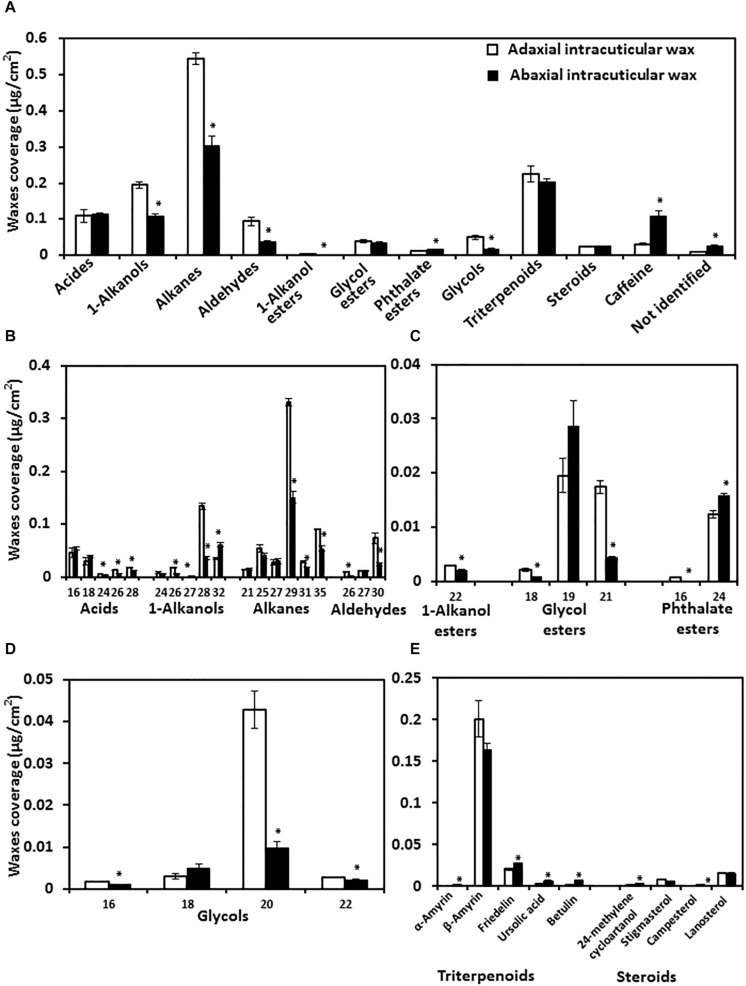
Wax lipid composition and chain length distribution of adaxial and abaxial intracuticular waxes. **(A)** Intracuticular wax compositions. **(B)** Chain length distributions of fatty acids and their derivatives. **(C)** Chain length distributions of esters. **(D)** Chain length distributions of glycols. **(E)** Chain length distributions of triterpenoids and steroids. Data were expressed as mean ± standard error (*n* = 4). *Statistically significant (*p* < 0.05).

To understand the chemical base of the transpiration difference, the composition of adaxial and abaxial IWs was analyzed. We found that the adaxial IWs showed higher coverage in very long chain fatty acids (VLCFAs), 1-alkanol esters, and glycols than that of abaxial IWs. Interestingly, caffeine was detected from the cuticle, and more enriched in the abaxial IW than the adaxial IWs (Figure 6A). The coverage of triterpenoids and steroids were similar between adaxial and abaxial IWs (Figures 6A,D).

The chain length distributions were also compared. Adaxial IWs were more enriched with longer-chain fatty acids (C24–C28), alkanes (C29, C31, and C35), 1-alkanols (C26 and C28), and glycol esters (C18 and C21) compared with abaxial IWs (Figures 6A,C).

The total glycol content of adaxial IWs was significantly higher than that of abaxial IWs, and mostly contained C20 and small portions of C16 and C22 molecules (Figure 6D). For triterpenoids and steroids, although their total contents were similar (Figure 6A), individual terpenoid component still showed large variations between adaxial and abaxial IWs (Figure 6E).

## Discussion

### The Assumptions, Limitations, and Advantages of the Proposed Method

The goal of this study is to measure transpiration rates from excised leaf; thus, the method of choice has to meet the following requirements: (1) the method should be able to specifically and consistently strip off EWs without affecting IWs; (2) the method should be non-destructive to the excised leaf. Jetter and Schäffer (2001) have demonstrated that gum arabic can specifically and consistently remove EWs by comparing with the most selective method that employs frozen glycerol as cryo-adhesive. Zeisler and Schreiber (2016) independently confirmed gum arabic’s specificity and consistency for EW removal. Jetter and Riederer (2016) applied gum arabic to remove EWs from eight plant species and located the transpiration barriers. We performed SEM and TEM observation to demonstrate its applicability to tea leaves; our data shown in Figure 1 were in accordance with previous reports (Jetter and Schäffer, 2001; Jetter and Riederer, 2016; Zeisler and Schreiber, 2016). Based on these data, we believe that gum arabic is the best fit for this research objective. Compared to other adhesives that often require organic solvents, gum arabic can be easily dissolved in water and thus avoid the possible side effects of organic solvent on IWs.

Several alternative methods in the literature have been used to remove EWs; they are either cryo-adhesive-based or polymer-based mechanical wax removal. Glycerol or water was commonly used as cryo-adhesive. Due to its destructive nature, cryo-adhesive-based wax removal method does not fit the described method. For polymer-based mechanical wax removal, collodion, cellulose acetate, and gum arabic were widely used (Haas and Rentschler, 1984; Silcox and Holloway, 1986; Jetter and Schäffer, 2001). Zeisler and Schreiber (2016) demonstrated that the organic solvent acetone used to dissolve cellulose acetate affected the transpiration barrier; thus, cellulose acetate was out of our consideration. Interestingly, the authors demonstrated that collodion and gum arabic are equally efficient to remove EWs; in addition, the collodion solvent (diethyl ether:ethanol) did not show side effects on IWs. These observations suggested that collodion could be an alternative EW removal agent. Compared to gum arabic, collodion shows several advantages for EW removal: (1) it can be conveniently applied to the CM surface; (2) solvent evaporation and the polymer film formation are fast (∼30 s) and thus can speed up the experiment and save time; (3) it does not damage CMs. In contrast, the water used to dissolve gum arabic evaporates much slower and takes about 1 h to form polymer film. In addition, gum arabic is not suitable to remove EWs from CMs. Although collodion has been demonstrated to be working well to remove EWs from the adaxial surface (Zeisler and Schreiber, 2016), it remains unclear if it is applicable to remove abaxial EWs; the presence of stomata on abaxial surface may affect collodion’s applicability on this surface.

Vaseline is a mixture of semi-solid non-polar hydrophobic hydrocarbon and insoluble in water, and it possesses excellent coating properties and is widely used as an ingredient in skin lotions and cosmetics by reducing skin moisture loss. In this study, we tested the effectiveness of vaseline in reducing leaf water loss. We found that vaseline application can reduce more than 70% leaf water loss (Figure 3). Thus, vaseline diffusion coefficient factor (k) was introduced in formula IV to XI to consider this leakage. k reflects the proportion of water loss through the applied vaseline film and should be affected by the thickness and uniformity of the vaseline film. During our experiment, care was taken to ensure that vaseline was evenly coated on both surfaces, and reproducible k was obtained in our experiments. As we mentioned above, k essentially reflects the physicochemical characteristics of the vaseline itself regardless where it is applied; theoretically, k should be stable once a consistent film was applied. However, during the 6-h post-excision, k increased from 0.21 to 0.29 (Table 1); this mild increase is mainly due to the way how it is calculated. We demonstrated that the gradual decrease of transpiration rates from the control leaves rather than from sealed leaves is responsible for the variation of k, the gradual decrease of transpiration rates from the control could be driven by the gradual stomata closure during post-excision (Figure 5A). As the data showed in Table 1, slight variation of k during post-excision had a large influence on the calculation of transpiration rates; we believe that the k value of the first hour should be used, and the transpiration rates at the first hour post-excision should be more accurate. One unresolved question is that if vaseline may interact with waxes, cutin and other lipids present in the cuticle even dissolve and diffuse in the cuticle due to its non-polar nature, these interactions consequently could alter cuticle structure and functionality. Although we can’t exclude these possibilities, considering that the transpiration rates from vaseline-coated leaves were kept constant during post-excision (Figure 5A), we tend to believe that vaseline application did not significantly interfere this proposed method.

The proposed method has two major limitations: (1) It does not apply to the leaves covered with trichomes, since gum arabic application will damage trichomes and cause uncontrolled water loss. In *C. sinensis* cv *Fuyun 6*, trichomes are present on the abaxial surfaces of tender leaves and shed off with leaf maturation. Thus, in this study, the mature fourth leaf was used. (2) It requires multi-step simultaneous leaf pretreatments.

Compared to other transpiration measurement methods, the proposed method has two advantages: (1) intact leaves can be directly used for transpiration rate measurement, and the obtained results are more relevant to leaf transpiration *in planta*. In contrast, other reported methods require the isolation of intact CMs, which is labor-intensive and time-consuming. (2) The proposed method can obtain transpiration rates from both leaf surfaces at the same time, and residual stomatal transpiration can also be estimated. This will offer us an integrated image about leaf transpiration.

### Residual Stomatal Transpiration Calculation

Accurate estimation of residual stomatal transpiration is essential for the calculation of abaxial cuticular transpiration, which raise the question if gum arabic stripping on abaxial surface affects stomatal closing. Here, three lines of evidences suggested that this is not the case: (1) stomata structure was kept intact after gum arabic stripping (Figures 1D,E); (2) gum arabic stripping to leaf abaxial surface did not affect ABA-promoted stomata closure (Figure 2); (3) the residual stomata transpiration rate from gum arabic-stripped leaves was similar to that of control leaves (Figure 5A).

Generally, stomata show variable response to ABA-promoted closing; this will lead to relatively large variations in leaf transpiration measurement. However, our transpiration data presented in Figures 2 and 3 showed small standard error bars; this may be attributed to the multi-step pretreatment procedures: (1) leaves were fully water equilibrated before experiment; it is well known that leaf water contents have a large influence on stomata transpiration; (2) leaves at the same developmental stage (the fourth leaf) were used; it is well-established that tender leaf and mature leaf show large variations in leaf water contents and transpiration rates; (3) the leaves were dark adapted overnight before ABA treatment; it is well known that dark promotes stomata closure. These data highlight the importance of the multi-step pretreatment procedures described in this proposed method.

If ABA treatment can make full stomata closure, then the 1st h transpiration difference between control leaves and ABA-treated leaves would accurately represent the residual stomatal transpiration. However, 50 μM ABA treatment did not make stomata fully close (Figures 5B,C). In another pretrial, 100 μM ABA was applied, but stomata aperture was similar to 50 μM ABA treatment. These observations suggested that for fully water equilibrated leaves, stomata remain partially open even after overnight dark adaptation; ABA application alone cannot make stomata close fully. However, at 5-h post-excision, stomata were mostly closed (Figures 5B,C); this is likely promoted by the decreasing leaf turgor pressure and the enhanced ABA synthesis during post-excision. Thus, the transpiration difference between 1st h and 5th h post-excision of the control leaf can be used to calculate stomatal transpiration. Considering that, even without ABA pretreatment, stomata still come to close fully at some time point of post-excision, this raises the question about the necessity of ABA pretreatment. We tend to believe that it is not plausible and not necessary through ABA application to obtain maximal reduction in stomatal transpiration, but ABA treatment can reduce sample variations; thus, it is helpful to obtain consistent reduction in stomatal transpiration. It is anticipated that the transpiration rate at 5th h post-excision could be lower than that of 1st h post-excision. Hence, the actual residual stomatal transpiration could be overestimated by this proposed method; accordingly, the abaxial cuticular transpiration was underestimated. Even so, the abaxial cuticular transpiration rate was still 50% higher than the adaxial cuticular transpiration rate (Table 1). Thereby, our main conclusions should not be affected much by the overestimation of residual stomatal estimation. The stomatal transpiration estimation in the proposed method assumed that the residual stomatal transpiration after stomata closure is fully negligible. Burghardt and Riederer (2003) observed that the minimum conductance of *Hedera helix* was threefold higher than the cuticular permeance; in contrast, the minimum conductance from the other four plant species was similar to their cuticular permeance. Based on these data, the authors suggested that the residual stomatal transpiration after full stomata closure was negligible for most plant species except *H. helix*. However, from the stated methods, the cuticular permeance was measured from adaxial cuticle, while the minimum conductance was measured from both adaxial and abaxial surfaces. An alternative explanation why minimum conductance of *H. helix* was threefold higher than the cuticular permeance could be due to its abaxial cuticular conductance much higher than that of its adaxial cuticular conductance; this will lead to a higher average value (so called “minimum conductance” by authors) than the adaxial cuticular permeance (so called “cuticular permeance” by authors). To further support this explanation, Šantrůček et al. (2004) demonstrated that for *H. helix* leaves, abaxial cuticular transpiration was about 11 times higher than that of the adaxial cuticular transpiration. These data suggested that after full stomata closure, residual stomata transpiration was negligible for all five plant species tested, and this likely is true for tea leaves. Thus, stomatal transpiration can be reliably estimated by using the transpiration difference before and after full stomata closure of control leaves.

### Tea Leaf Adaxial and Abaxial Surfaces Show Different Transpiration Barrier Organization

Zeisler and Schreiber (2016) and Zeisler et al. (2018) studied 10 different plant species including tea tree and concluded that the adaxial EWs were not the main cuticular transpiration barrier, and our data were in accordance with that conclusion. In addition, unlike the adaxial EWs, the abaxial EWs contributed significantly to the cuticular transpiration barrier (Figures 4, 5). The adaxial EWs stripped off by gum arabic was only slightly higher than that of abaxial EWs (Supplementary Table S1), suggesting that gum arabic had similar efficiencies in removing EWs in adaxial as in abaxial surfaces; the lower effect on transpiration induced by gum arabic when applied to adaxial surface was unlikely due to less EWs removed compared to abaxial surfaces. At the tissue level, adaxial IWs and abaxial EWs constituted the major leaf transpiration barriers, while adaxial EWs and abaxial IWs contribute negligibly to leaf transpiration barrier. We also demonstrated that abaxial cuticular transpiration rate was 50% higher than adaxial cuticular transpiration rate (Table 1); this was in accordance with the report of Šantrůček et al. (2004) even though different methods were applied by these two studies.

### Relationships Between Wax Composition and Cuticular Transpiration

Cuticle is a highly heterogeneous structure and formed by an array of compounds with different physicochemical properties (Fernández et al., 2017). Khayet and Fernández (2012) demonstrated that alkanes are fully apolar molecules, whereas molecules containing oxygen in their functional groups (e.g., alcohols, acids, ketones, or ester bonds) had some degree of polarity and H-bonding interactions. Thus, it is anticipated that wax composition would affect cuticular transpiration properties. After comparing transpiration in combination with wax analysis from eight different plant species, Jetter and Riederer (2016) concluded that transpiration barrier is associated mainly with VLCFA derivatives. Here, we found that transpiration rate from abaxial IWs was threefold higher than that of adaxial IWs (Table 1); this provided an independent system to reexamine that conclusion (Jetter and Riederer, 2016). We found that some compounds from adaxial IWs, including VLCFAs (1-alkanols, alkanes, and aldehydes), 1-alkanol esters, and glycols, showed higher contents than that of abaxial IWs (Figure 6A). Thus, our data from tea leaves were in accordance to that conclusion (Jetter and Riederer, 2016). Currently, there are few integrative studies on the internal nature of cuticle (Viougeas et al., 1995; Yeats et al., 2012); it remains largely unknown if there are potential cross-links and/or molecular assemblies between cuticular chemical constituents (Kunst and Samuels, 2009). In addition, given the major structural and chemical variability of plant cuticles, more research was still needed before a valid general cuticle model could be established for all species or organs (Fernández et al., 2017). Interestingly, caffeine was detected from tea IWs as well as EWs, and more enriched from abaxial IWs compared to adaxial IWs. Currently, it remains unclear if caffeine has any contribution to cuticle barrier properties. Previous studies demonstrated that caffeine has potent antipathogenic and antiherbivore activities (Uefuji et al., 2005; Kim and Sano, 2008; Wang et al., 2016), suggesting that caffeine is potentially involved in cuticle’s defense against pathogens and herbivores rather than a contributor to transpiration barrier.

## Conclusion

We established a new method that allows simultaneous measurement of cuticular transpiration rates from individual leaf surface. By applying this method, we demonstrated that the adaxial IWs and the abaxial EWs constitute the major cuticular transpiration barriers; the abaxial cuticular transpiration rate was 50% higher than that of adaxial surface. These new findings would facilitate the identification of key factors that delineate leaf barrier properties.

## Data Availability Statement

All data generated or analyzed for this study are included in the article/Supplementary Material.

## Author Contributions

YZ and XC carried out experiments and prepared SEM and TEM samples. YZ analyzed the wax lipids with ZD, WZ, and ZC. CC provided the study materials. MC and WS designed the experiments. MC, YZ, and ZC wrote the manuscript. AD helped with manuscript editing. All authors contributed to manuscript revision, and read and approved the submitted version.

## Conflict of Interest

The authors declare that the research was conducted in the absence of any commercial or financial relationships that could be construed as a potential conflict of interest.

## Abbreviations

ABA: abscisic acid
BSTFA: *N*,*O*-bis(trimethylsilyl)-trifluoroacetamide
SEM: scanning electron microscope
TEM: transmission electron microscope
VLCFAs: very long chain fatty acids.
